# The factory enters the fray: how mitochondrial protein trafficking shapes the host response to infection

**DOI:** 10.1128/jvi.01746-25

**Published:** 2026-09-14

**Authors:** Dustin C. Hancks, Steven F. Baker

**Affiliations:** 1 Department of Immunology, University of Texas Southwestern Medical Center 12334, Dallas, Texas, USA; 2 Department of Microbiology and Immunology, Stritch School of Medicine, Loyola University Chicago 12248, Maywood, Illinois, USA; New York University Department of Microbiology, New York, New York, USA

**Keywords:** mitochondria, viruses, protein localization, immunometabolism, adaptation

## Abstract

Beyond textbook functions in homeostatic metabolism, mitochondria are now recognized as central coordinators of cell-intrinsic and cell-extrinsic immune responses to infection. Directed trafficking of proteins and other molecules between mitochondria and the rest of the cell underlies a growing catalog of these activities. Some are pro-host; others are antagonized by viral effectors or co-opted by viruses entirely. How host and viral factors rewire the mitochondrial proteome during infection to shape these outcomes remains incompletely understood. The evolutionary history of this system adds another dimension: mitochondria retain biochemical signatures of their α-proteobacterial endosymbiotic origin, and ongoing co-evolution between viral, host, and mitochondrial genomes continues to shape the proteins that traffic to and from the organelle. Using published examples, we highlight general principles, mechanisms, and consequences of host and viral protein localization to and from the mitochondria. To support discovery, we present integrated gene lists identifying host mitochondrial factors with evidence for type I interferon stimulation, interactions with viral proteins, and signatures of positive selection. Together, these resources and the principles within offer a framework for understanding mitochondria not as passive metabolic machinery but as actively contested cellular territory whose protein composition is continuously negotiated between the host and the pathogen.

## INTRODUCTION

### Overview

Mitochondrial protein trafficking sits at the intersection of metabolism and immunity. Redistribution of host and viral proteins to and from the mitochondria underlies a wide range of infection outcomes, and the same molecular circuitry operates in non-infectious settings, including metabolic disease and cellular stress (1–3). Many pattern-recognition receptors (PRRs), first characterized as sensors of pathogen-derived nucleic acids, also detect structurally similar host-derived molecules produced during sterile stress or disease, including cancer (4–7). Conversely, immune functions discovered in non-infection contexts may not reflect a response’s primary evolutionary purpose. Viruses impose some of the strongest selective pressures known (8), and many stress-response pathways, now recognized as broadly protective, may have originally evolved as antiviral adaptations. Contemporary protein distribution to and from the mitochondria was shaped by the domestication of mitochondria by cells and host-pathogen co-evolution. Understanding the identity of dual-localizing proteins and the mechanisms by which they are deployed has far-ranging implications in health and disease.

### Mitochondrial biology

**Fig 1:**
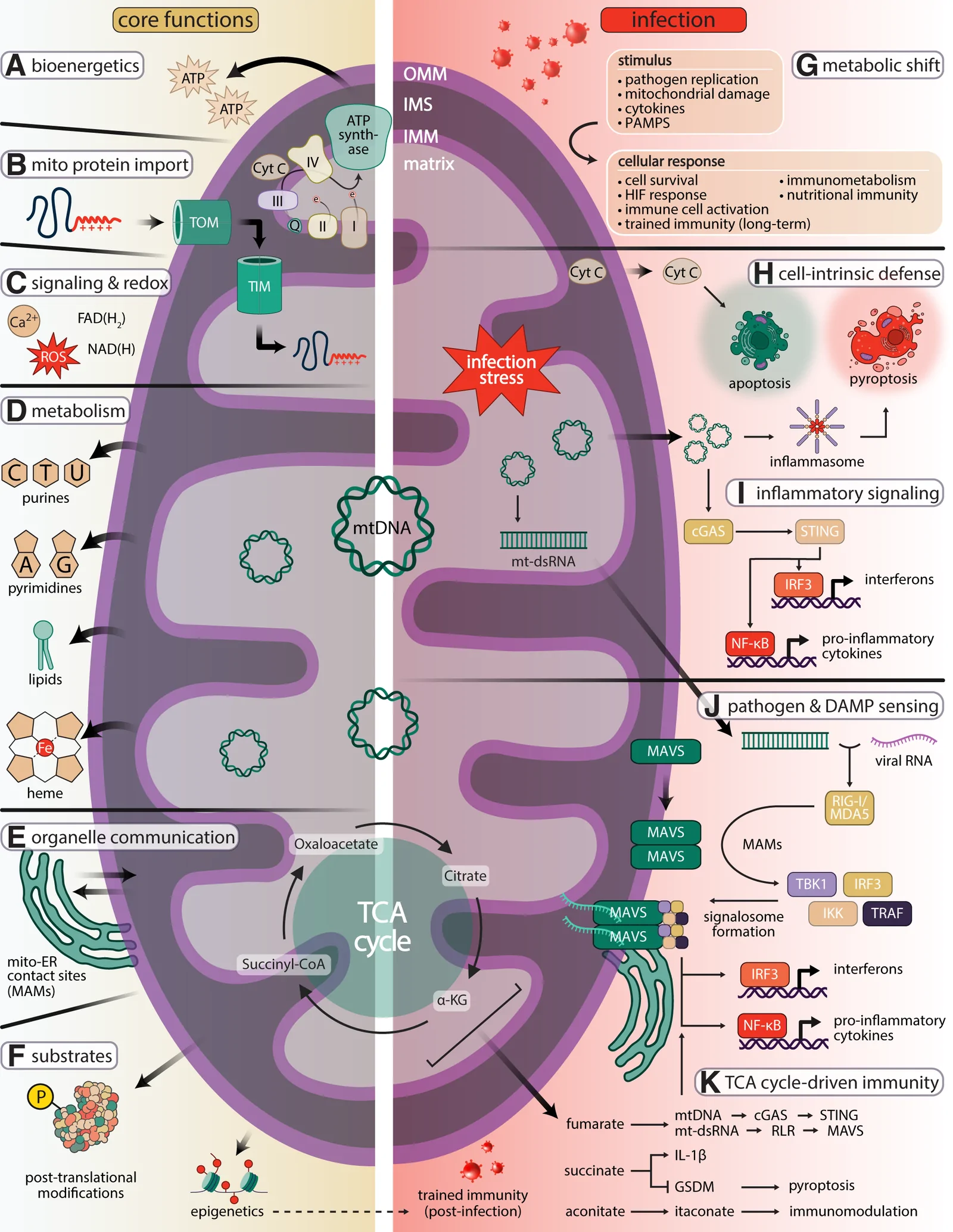
Known roles for mitochondria in homeostasis and immunity. Mitochondria play key roles in core cellular functions, the response to infectious agents like viruses, and stress adaptation. (**A–F**) Core functions for mitochondria in cell biology, and (**G–K**) roles for mitochondria and their activities during infection and the host response. (**A**) Bioenergetics. Mitochondria are essential producers of ATP via electron transport mediated by the electron transport chain (Complexes I–IV, ATP synthase, cytochrome c [cyt c], and ubiquinone [Q]). (**B**) Mitochondrial protein import. To carry out vital functions, mitochondria import proteins, which are encoded by the nuclear genome and translated in the cytoplasm. Many nuclear-encoded proteins encode targeting sequences like an N-terminal amphipathic helix. (**C**) Signaling and reduction and oxidation (REDOX). Mitochondria regulate the levels of molecules that maintain the REDOX balance in the cell and have roles in cell signaling, such as FAD(H_2_), NAD(H), and calcium (Ca^2+^). Reactive oxygen species (ROS), a byproduct of the electron transport chain Complexes I and III, impact the cellular REDOX state and act as signaling molecules. (**D**) Metabolism. Mitochondria contribute to many cellular metabolic pathways, including purine, pyrimidine, lipid, and heme metabolism. (**E**) Organelle communication. Mitochondria interact with other cellular organelles, and this cross-talk regulates not only mitochondrial activities but also functions of the interacting organelle. For instance, mitochondrial-associated ER membranes (MAMs) mark sites of mitochondrial division. (**F**) Substrates. Several mitochondrial-associated metabolites serve as substrates and co-factors in chemical reactions. ATP produced by the electron transport chain, for example, is used by kinases to phosphorylate proteins. Likewise, TCA cycle-associated metabolites, such as α-ketoglutarate (α-KG) and acetyl-CoA, which can be produced from citrate, are critical for epigenetic regulation. Specifically, these metabolites are used as co-factors and substrates in post-translational modifications of histones (i.e., methylation and acetylation). (**G**) Metabolic shift. Metabolic rewiring of infected, bystander, and immune-activated cells is a hallmark of pathogen infection and is induced by several stimuli. At the cellular and organismal levels, this metabolic reprogramming can result in both short-term and long-term changes (i.e., trained immunity). (**H**) Cell-intrinsic defense. In response to infection, stress, and damage, mitochondria release molecules into the cytoplasm that activate host defenses like cytochrome c, which can cue apoptosis, and mitochondrial (mtDNA), which can trigger the inflammasome, resulting in pyroptosis. (**I**) Inflammatory signaling. mtDNA released into the cytoplasm can activate the cGAS-STING pathway, ultimately leading to the production of type I interferons and cytokines mediated by the transcription factors IRF3 and NF-kB. (**J**) Pathogen and DAMP sensing. During viral replication, mitochondria activate immune responses by releasing danger-associated molecular patterns (DAMPs; mtDNA and ds-mtRNA) and by serving as a platform for pathogen recognition receptor signaling, as in the case of RIG-I-like receptors (RIG-I and MDA-5) and MAVS. Activation of RIG-I-like receptors leads to the formation of the MAVS signalosome, which involves the recruitment of cellular proteins (TBK1, IRF3, IKK, and TRAF) and cellular RNAs. In addition, interactions with the ER and mitochondria shape MAVS signaling. (**K**) TCA cycle-associated metabolites are involved in immune-related processes, including cGAS-STING, MAVS, and pyroptosis.

Beyond their canonical role in ATP production through oxidative phosphorylation, mitochondria are essential biosynthetic hubs responsible for the synthesis of iron-sulfur clusters, heme, and fatty acids (Fig. 1A through F; 9). To maintain cellular homeostasis, the energetic fitness of this network is rigorously surveilled through quality control mechanisms. Defective mitochondria or pieces thereof are selectively removed through mitophagy (10, 11), which links marked mitochondria with lysosomes for degradation, while more severe dysfunction can trigger mitochondrial stress responses or initiate programmed cell death (apoptosis) (9, 12). When the integrity of these double-membraned compartments is compromised, mitochondria can release mitochondrial damage-associated molecular patterns (DAMPs), such as immunostimulatory nucleic acids (mtDNA and mtRNA) and cardiolipin, which serve as “alarmins” to activate cellular innate immune responses (13–15).

Cellular organelles coordinate eukaryotic cell adaptability to environmental changes by integrating metabolic flux with external stimuli. Mitochondria are central to this plasticity, existing not as isolated “powerhouses” but as a dynamic, interconnected intracellular network that moves via cytoskeletal motors (16). This network is constantly remodeled through balanced cycles of fission and fusion, which regulate organelle size, shape, and constituents to meet the shifting environmental or cell-intrinsic cues (17). Notably, mitochondria interface and communicate with most other cellular organelles, including the endoplasmic reticulum (ER), peroxisomes, Golgi, and lipid droplets (18, 19). Mitochondria-associated ER membranes (MAMs) are sites of direct contact of mitochondria with the ER that are essential for marking the sites of mitochondrial fission (20). Mitochondria-peroxisome contacts are another important interface contributing to the regulation of mitochondrial ROS (21). This functional integration of structure, metabolism, and surveillance establishes mitochondria as a fundamental rheostat for both cell health and host defense.

From their evolutionary origin as α-proteobacterial endosymbionts, mitochondria have retained biochemical signatures, such as unmethylated CpG DNA and N-formylmethionine, that continue to blur the lines between cellular “self” and bacterial “non-self” (22–24). A single mitochondrion is defined by its unique double-membrane architecture: a relatively permeable outer mitochondrial membrane (OMM) and an extensively folded inner mitochondrial membrane (IMM) that forms cristae that maximize surface area for OXPHOS (25, 26). The matrix, or the mitochondrion core, houses the mitochondrial DNA (mtDNA) and the machinery required for its expression, while an electrochemical gradient established across the IMM powers the generation of ATP by respiratory chain complex members (27). Of the >1,100 proteins found within the mitochondria (28, 29), less than 1% are encoded by the 16.6 kb circular mtDNA genome. Thus, a massive influx of nuclear-encoded products is required (30).

The vast majority of mitochondrial proteins are actively imported through the translocases of the OMM and IMM (TOM and TIM, amino acid charge-specific complexes) and stabilized by molecular chaperones (Fig. 1B; 31). Within the organelle, functional plasticity is maintained through transcriptional and translational control, as well as the action of AAA (ATPases associated with diverse cellular activities) family proteases that scan and degrade misfolded or damaged proteins (9, 32, 33). This sophisticated import and surveillance machinery does not merely maintain metabolism but, together with mitophagy, acts as a critical checkpoint where the selective licensing or diversion of proteins can repurpose canonical mitochondrial factors for specialized roles in host defense (34). Understanding how proteins are differentially localized reveals how the cell leverages the “non-self” of mitochondria to orchestrate complex immune responses.

Proteins reach mitochondria through at least five distinct import mechanisms (35). Many contain hydrophobic or unstable domains that require chaperone delivery by HSP70 and HSP90 prior to import; upon sorting, these domains are removed, modified, or used to anchor the protein within mitochondrial membranes. The first two import mechanisms involve the insertion of OMM-bound proteins; the OMM is studded with single- and multi-spanning α-helical transmembrane proteins and β-barrel proteins, all of which are nuclear-encoded (36). β-barrel proteins enter mitochondria through the TOM complex, are stabilized by TIM chaperones within the mitochondrial intermembrane space (IMS), and are then inserted into the OMM by the sorting and assembly machinery (SAM). α-Helical OMM membrane proteins, by contrast, do not enter through the TOM40 channel and have different entry mechanisms depending on their topology. It remains unclear how tail-anchored proteins with C-terminal membrane domains are inserted into the OM, but signal-anchored proteins (N-terminal membrane domain) require TTC1, multi-pass proteins require the NAC complex, and all three classes require the MTCH2 insertase (37). Third, and the most well-characterized, is through the presequence pathway, whereby ~60% of all mitochondrial proteins enter via their N-terminal mitochondria-targeting signal (MTS) (38). These presequences are positively charged and form amphipathic α-helices. Upon arrival at the OM, presequences are recognized by TOM20 and TOM22, and pass through TOM40 channels. Once within the IMS, MTS-containing proteins bound for the matrix engage with TIM23 and are motored through the IMM via a mitochondrial HSP70-containing complex in the matrix. Presequence-containing proteins can be embedded in the IM if they contain a hydrophobic sorting signal downstream of the MTS, which leads to arrest at TIM23 and subsequent release to the IMM or import to the matrix and retrograde insertion into the IM via the OXA insertase. Fourth, the carrier pathway shuttles IMM-bound multi-spanning hydrophobic proteins to engage with TOM70 at the OM and translocate through TOM40 channels. In the IMS, these proteins are stabilized by TIM chaperones, preventing their aggregation, and then finally use the TIM22 complex to insert them into the IM (35). Finally, characteristic cysteine motif-containing proteins can pass through the TOM40 channel, where the cysteines are oxidized by the mitochondrial IMS import and assembly (MIA) system to form disulfide bonds (36). Notably, membrane potential, which can be disrupted by perturbation to the electron transport chain, stress (e.g., infection), and mitochondrial damage, is vital for the import of many mitochondrial proteins (39–42). The diversity of topologies that license proteins for mitochondrial delivery creates ample opportunity for infection-mediated transcriptional or post-translational changes to modify protein mitochondrial homing.

### Mitochondria and viruses

Mitochondrial activities intersect with viral infection in two main ways: they benefit the host, or they are co-opted by the virus (Fig. 1G through K; 24, 43–47). Viruses may also perturb mitochondrial functions without directly co-opting them, disrupting host defenses as a secondary consequence of replication (43, 48, 49). Host mitochondrial activities influence cell-intrinsic and cell-extrinsic responses, including PRR signaling and inflammation, respectively (50, 51). A paradigmatic example of cell-intrinsic defense is cytochrome c release from mitochondria, which catalyzes apoptosome formation and links an essential housekeeping function directly to antiviral cell death (52). The broader principle that transfer of molecules from mitochondria to the cytosol drives fundamental immune responses and that viruses subvert this process continues to generate important discoveries (45, 53–56). Cytosolic release of mtDNA and mitochondrial double-stranded RNA (mtdsRNA) are now among the most studied mitochondrial danger signals. While the TCA cycle has key homeostatic functions, several TCA cycle-associated metabolites, including fumarate (57, 58), succinate (59, 60), and itaconate (61, 62), which is derived from aconitate, have several important roles in regulating immunity.

The mitochondrial proteome is not fixed. Regulation and localization of host factors, including mitochondrial proteins, differ by cell type and cell state (28, 63–66). While most nucleated cells contain mitochondria, the proteins within them vary considerably. MitoCarta, which cataloged mitochondria-localizing proteins across a panel of normal tissues, found that there was only ~75% overlap between any two tissues (28, 67, 68). Comparable analyses in virally infected cells are largely absent but could resolve conflicting reports of viral protein localization and reveal infection-specific shifts in mitochondrial composition. Additional variables likely influencing the contextual mitochondrial proteome include cell cycle, metabolic state, and oxygen availability.

#### Mitochondria and host responses

Metabolic reprogramming of dedicated immune cells (macrophages, T cells, and others) is the best-characterized connection linking mitochondrial function to extrinsic host defense (44, 60, 69, 70). This interplay between metabolism and systemic immunity is broadly termed immunometabolism (71). An exemplar of immunometabolism is the glycolytic shift in macrophages activated by LPS, which drives M1 polarization (72). Many immune cell types rewire their metabolism in response to cytokines and pathogen-associated molecular patterns (PAMPs) (44), and the downstream consequences of this reprogramming, including epigenetics, transcription, and protein translation, remain incompletely understood. Less appreciated is that non-immune cells, which often represent the primary sites of viral infection, may undergo analogous metabolic changes that contribute to cell-intrinsic antiviral responses (66, 73).

How metabolic shifts unfold *in vivo*, especially in the context of metabolic comorbidities, is understudied compared with the depth of characterization in tissue culture (74–89). Most work has focused on how discrete signals such as cytokines drive metabolic change, but the reciprocal question is equally important: how do metabolic states themselves modulate immune responses (47, 74, 83, 88)? During infection, one possibility is that pre-existing metabolic states prime or dampen immunity as secondary contextual cues. This model is consistent with the disproportionately poor infection outcomes observed in patients with metabolic comorbidities (66, 88, 90). Trained immunity results from metabolic reprogramming (91–93). Well-studied in innate immune cells, trained immunity arises when prior immune activation drives epigenetic changes that later affect the magnitude of immune responses within those cells to subsequent stimulation. Studies of trained immunity revealed that infection history impacts not only adaptive but also innate immunity.

Molecular flux into mitochondria during infection is equally relevant but far less studied. Mitochondrial uptake of metabolic intermediates such as pyruvate and glutamine is critical for homeostasis and is perturbed in human disease (94–96). More directly, mitochondrial dNTP import is regulated by the interferon-stimulated gene (ISG) *CMPK2* and the antiviral factor SAMHD1 (97–101). dNTP flux drives new mtDNA synthesis, which is required for NLRP3 inflammasome activation (102, 103). Together, these findings provide evidence that mitochondrial import, and not just export, contributes to host defense.

Mitochondrial antiviral signaling protein (MAVS) is a critical and widely studied adaptor protein for RIG-I-like receptor (RLR) signaling, which initiates a cascade to induce IFN upon PRR detection (4, 50, 104–107). MAVS signaling occurs at MAMs, which was demonstrated by its antagonism by the HCV NS3/4A protease (108). MAMs are dynamic during infection, and identifying host and viral factors trafficking to and from MAMs will likely yield novel infection determinants and insights into mitochondrial regulation (109). Additionally, host factors may have context-dependency and function differently within organelles that contact mitochondria. For example, MAVS signaling occurs both on mitochondria and peroxisomes (110). MAVS signaling on peroxisomes accounts for the early response during infection, followed by MAVS signaling on mitochondria. Altogether, observations made through studying MAVS demonstrate that interactions of mitochondria with other cellular compartments represent a critical battleground for host defense against pathogens. New imaging modalities will likely yield valuable insights into how this cross-talk is mediated, perhaps through molecular trafficking.

**Fig 2:**
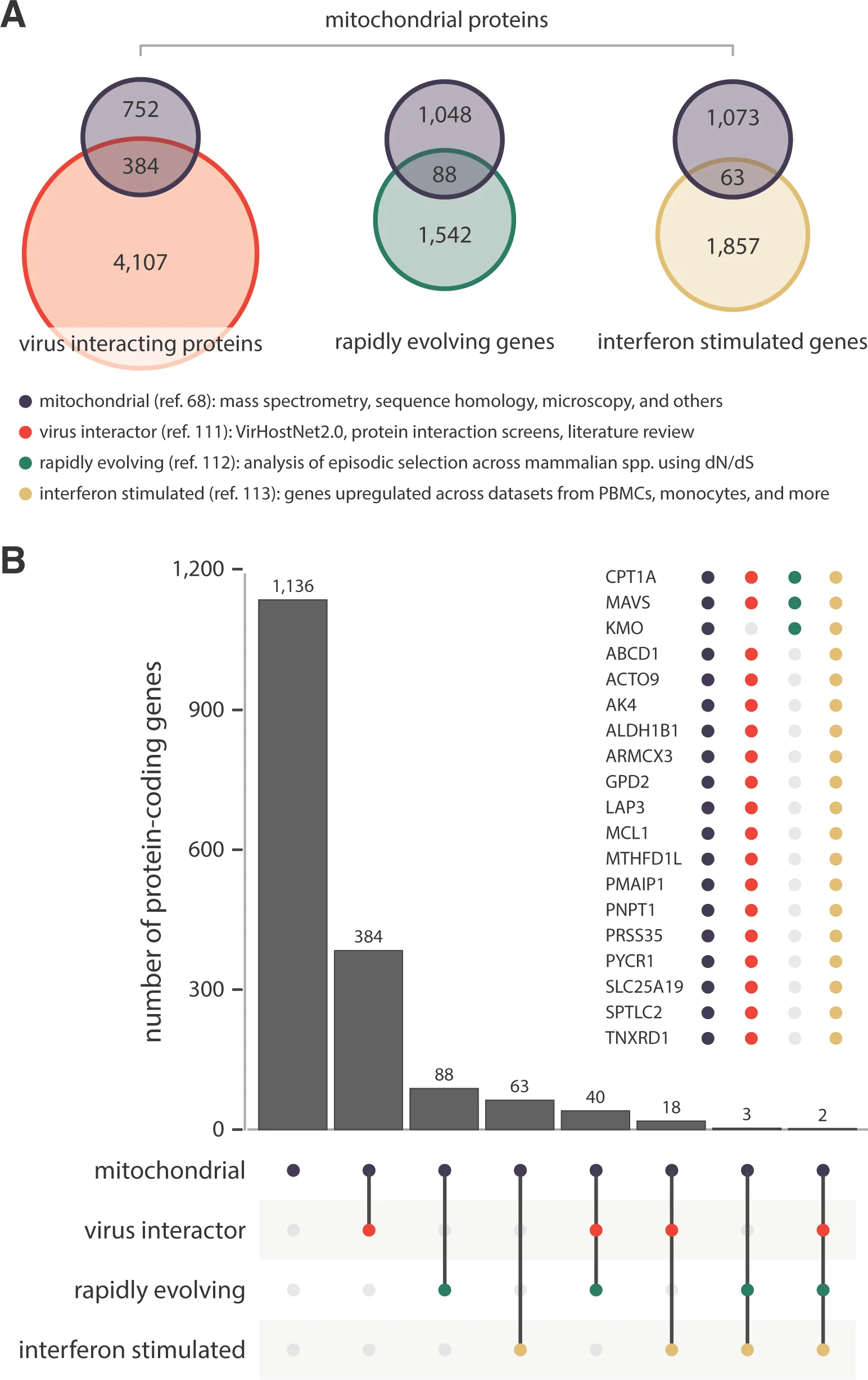
Mitochondrial proteins implicated in immunity. (**A**) Venn diagram depicting one-way overlap between mitochondrial proteins and indicated protein and gene sets. (**B**) One-, two-, and three-way comparisons across lists. The number of protein-coding genes identified in each gene set is indicated. Inset depicts the results from the three rightmost columns on the bar chart. The full lists are available in Table S1.

To identify mitochondrial proteins with putative immune functions, one approach is to cross-compare the organellar proteome with lists of immune-related genes (Fig. 2). We perform a cross-comparison here using lists of mitochondrial proteins, virus-interacting proteins, rapidly evolving genes, and ISGs (68, 111–113). 1,136 mitochondrial proteins were identified by integrating purified mitochondria and APEX proximity mitochondrial mass spectrometry data, mitochondrial-targeting signal predictions, homology to yeast mitochondrial proteins, mRNA co-expression analyses, and manual review of microscopy and fractionation experiments (68). In total, 4,491 virus-interacting proteins were determined using a database (VirHostNet2.0; 2,614 proteins) plus 14 other recent screening studies, excluding yeast two-hybrid studies, and additional literature searches (111, 114). Finally, 1,630 rapidly evolving genes were determined using statistical models (BS-REL and BUSTED) to detect episodic selection, which identifies genes where the rate of amino acid changes (dN) is significantly higher than the rate of silent mutations (dS) within codons (112). Lastly, 1,920 ISGs were included if showing a significant increase in expression (fold change ≥ 2) across multiple independent interferon-stimulation microarray and RNA-seq data sets from PBMCs, monocytes, monocyte-derived macrophages, and THP-1 cells or a fold change ≥ 3 if identified in only one study (113). Of the 1,136 mitochondrial proteins, 384 have been identified as virus-interacting proteins, 88 mitochondrial proteins are encoded by genes undergoing rapid evolution and as such are implicated in host-pathogen conflict, and 63 are encoded by ISGs (Fig. 2A). There are only two genes that are members of all four lists (mitochondrial, virus interactor, rapidly evolving, and ISG): carnitine palmitoyltransferase 1A (CPT1A) and MAVS (Fig. 2B). CPT1A, in contrast to MAVS, has yet to be evaluated in the context of host defense. CPT1A is a fatty acid transporter whose mutation is associated with hypoglycemia and poor prognoses, particularly in indigenous Arctic populations (115, 116). It should be noted, however, that this approach of identifying genes that belong to multiple lists is a means for hypothesis generation and that further validation is required. Additionally, these lists were generated using multiple experimental systems and species, and as such do not represent a comprehensive catalog of immune-related mitochondrial factors. This approach or others aimed to uncover moonlighting mitochondrial proteins can more intentionally unearth hidden facets of immunity.

## REGULATORY AND EVOLUTIONARY CONTROL OF MITOCHONDRIAL TARGETING

**Fig 3:**
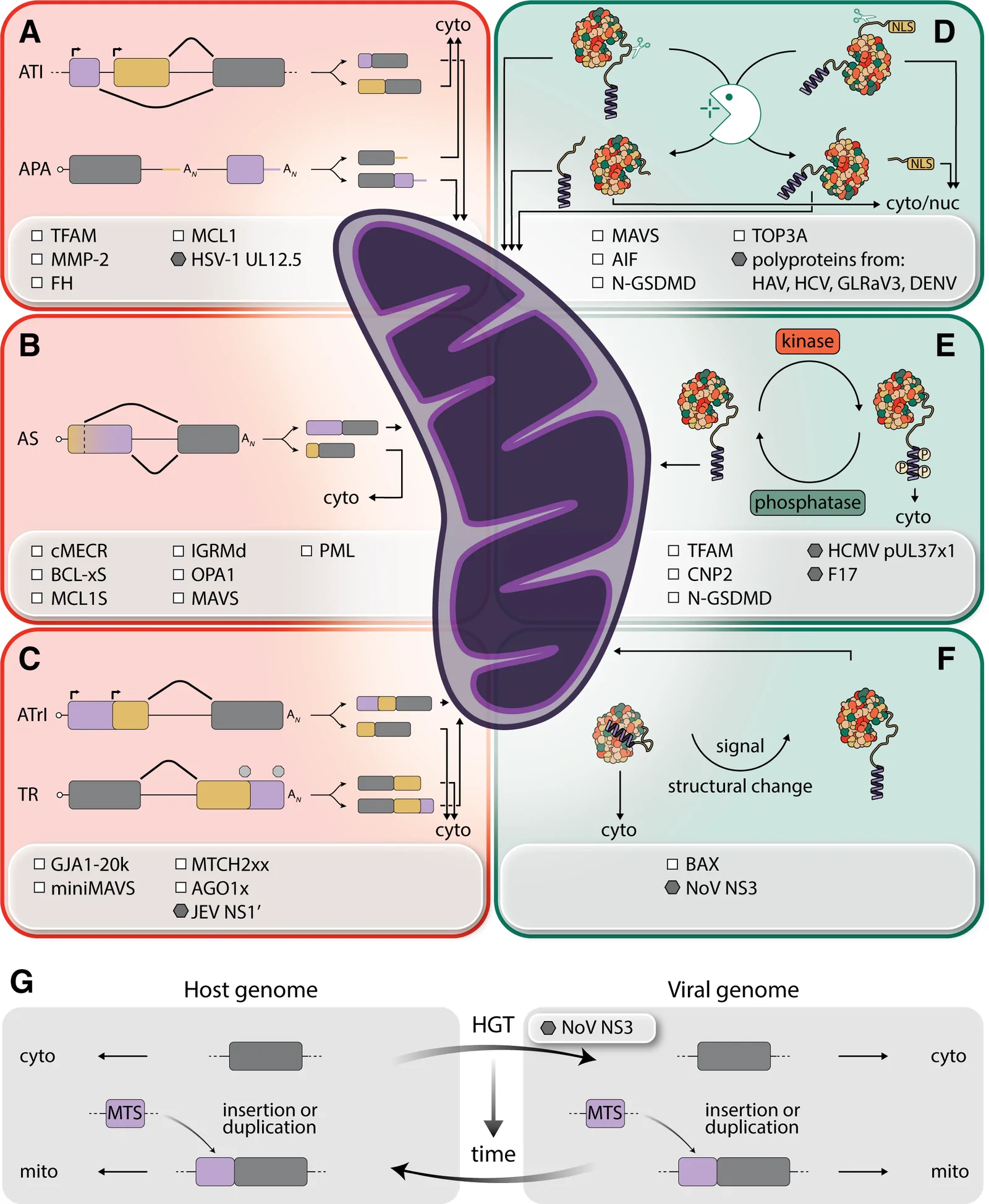
Molecular roads that lead to the mitochondria. Schematic representation of select infection-sensitive transcriptional, translational, post-translational, and evolutionary mechanisms bestowing mitochondrial localization to protein products. Mitochondrial targeting sequence indicated as magenta exon or α-helix. (**A**) Alternative transcription initiation or alternative polyadenylation sequences change the 5′ or 3′ transcript untranslated region. (**B**) Alternative mRNA splicing incorporates unique pieces or entire exons or introns, generating a variety of transcripts with diverse coding potential. (**C**) Alternative translation initiation and translational recoding via stop codon readthrough or frameshifting by ribosomes synthesizes proteins with unique N- and C-termini from the same transcript. (**D**) Protease-mediated cleavage can separate pro-migratory (left) or anti-migratory (right) signals from proteins to shape localization. (**E**) Post-translational modifications, for example, phosphorylation, reversibly mask localization signals. (**F**) Structural changes promote trafficking to mitochondria by making localization signals accessible. (**G**) Evolutionary sequence gains and losses encoding mitochondrial-targeting sequences or transmembrane domains rewire protein functions across species in a single mutational step. Adaptation was mediated by horizontal gene transfer to and from viral genomes and likely occurred during mitochondrial domestication. Insets within subsections show host (open square) and viral (filled hexagon) genes discussed in this review, with citations in body text. ATI, alternative transcription initiation; APA, alternative polyadenylation; AS, alternative splicing; ATrI, alternative translation initiation; TR, translational recoding; NLS, nuclear localization sequence; HGT, horizontal gene transfer; MTS, mitochondrial targeting signal; TM, transmembrane domain; cyto, cytoplasm; nuc, nucleus; mito, mitochondria.

The mechanisms controlling dual mitochondrial localization span transcription, translation, and post-translational modification (PTM), each representing an independent axis along which host and viral proteins evolve (Fig. 3). Importantly, selection acts on entire regulatory circuits rather than individual components. A phosphorylation-dependent localization switch, for instance, simultaneously places the modifiable residue, the responsible kinase, and that kinase’s own expression and localization under selection pressure. The host therefore has multiple independent knobs to tune localization outcomes, and viruses face a correspondingly multi-layered target to subvert or exploit.

### Transcriptional and translational control of localization

In response to pathogen detection, host cells deploy coordinated gene expression changes to limit replication and promote survival. A hallmark response is transcriptional upregulation of ISGs, which can individually increase by factors of 2-fold to over 2,000-fold (117). Beyond transcript abundance, infection also reshapes transcript quality. Many genes encode multiple isoforms through alternative transcription initiation, splicing, polyadenylation, or translational recoding, and the encoded protein isoforms frequently differ in their subcellular localization. As presequences are encoded at the 5′ end of the transcripts, any mechanism that modifies the 5′ region or the N-terminus of a protein has direct potential to alter mitochondrial targeting.

#### Alternative transcription initiation (ATI)

The transcription start site (TSS) defines the first transcribed nucleotide in a gene and is functionally linked to the surrounding core promoter region (118). Over 50% of human genes contain more than one promoter, and on average, four TSSs are annotated per gene (119, 120). ATI refers to the use of non-canonical TSSs and carries multiple functional consequences: it can shorten or lengthen the 5′ untranslated region (UTR), affecting transcript stability, or alter the coding sequence to produce N-terminally truncated or extended proteins. ATI adds regulatory capacity to genes and contributes to developmental, cell type, and tissue-specific expression patterns (121). Most alternative initiation events are not adaptive, arising predominantly from molecular errors (122). Nevertheless, because presequences are encoded near the 5′ end of transcripts, ATI has clear potential to modify mitochondrial targeting (Fig. 3A).

The UL12 and UL12.5 proteins of herpes simplex virus 1 (HSV-1) illustrate how ATI can govern mitochondrial targeting and viral lifecycle control. The *UL12* locus contains two promoters, and its expression promotes degradation of host mtDNA early in infection (123–126). UL12.5, produced as an ATI event from the downstream promoter, encodes an MTS and recruits host endonucleases ENDOG and EXOG to deplete mtDNA (127, 128). HSV-1 can establish latent infection in neurons, and lytic reactivation from this state requires UL12.5. Its expression in latently infected neurons triggers a pro-inflammatory cascade driven by mtDNA detection that is required for lytic cycle re-activation (129). A single ATI event thus connects mitochondrial targeting to the switch between latent and lytic infection.

Host mitochondrial localization can similarly be controlled by ATI. Mitochondrial transcription factor A (TFAM) is a multifunctional nuclear-encoded protein that serves as a mtDNA nucleoid component, playing major roles in maintaining mtDNA copy number and driving mtDNA transcription (130–132). TFAM produces non-mitochondrial isoforms through ATI specifically in human testis (133), and subsequent work confirmed that such isoforms can arise from transcript variation more broadly (134). In a landmark study, Lee et al. demonstrated that mislocalized TFAM in sperm cells underlies the lack of paternal mtDNA transmission (135). TFAM is also regulated by post-translational mechanisms discussed in “Post-translational modifications and mitochondrial trafficking,” below. In heterozygous mice, TFAM haploinsufficiency drives immunopathology through cytosolic mtDNA accumulation and cGAS/STING activation (136), raising the possibility that TFAM-dependent localization shifts could similarly amplify innate immune responses during infection. Two additional enzymes with cytosolic ATI isoforms, fumarate hydratase (FH) and matrix metalloproteinase 2 (MMP-2), also warrant attention (137, 138). FH regulates mtRNA- and mtDNA-mediated innate immune stimulation, while an oxidative-stress-induced MMP-2 isoform localizes to mitochondria and activates pro-inflammatory pathways (57, 58, 138). Neither has yet been directly implicated in infection, but their roles in sterile inflammation make them compelling candidates.

#### Alternative polyadenylation (APA)

During co-transcriptional mRNA processing, the 3′ end is cleaved and polyadenylated by a multiprotein core complex with many associated factors (139). APA can occur terminally or internally, generating shorter or longer 3′ UTRs that affect RNA localization or stability, or internally within introns, truncating the open reading frame (ORF) and producing C-terminally shortened proteins. Myeloid cell leukemia 1 (MCL1), a member of the B cell lymphoma 2 (BCL-2) family of proteins that resides on the OMM, exemplifies both the immunological relevance of APA and its susceptibility to viral perturbation. BCL-2 family proteins act as guardians, initiators, or executioners of apoptosis through the regulation of mitochondrial outer membrane permeabilization (MOMP) (140). MCL1 is an anti-apoptotic guardian protein, and infections with influenza A, corona, herpes, and flaviviruses all result in MCL1 expression differences that tune apoptotic induction (141). T cells make two MCL1 transcripts through APA, pA1 and pA2, differing in 3′ UTR length. T cell activation increases pA1 accumulation, boosting MCL1 protein abundance, while simultaneously inducing microRNA-17 (miR-17) to suppress pA2 (142). The scope of infection-driven APA extends well beyond MCL1. During vesicular stomatits virus (VSV) and influenza A virus infection, global APA changes correlate with altered cleavage and polyadenylation specificity factor (CPSF) abundance, although the direction of change differs: VSV shortens 3′ UTRs on average, while influenza A virus lengthens them (143, 144).

#### Alternative mRNA splicing (AS)

Pre-mRNA splicing is carried out co-transcriptionally by the spliceosome, which removes non-coding introns from nascent transcripts (145). AS arises when exons or introns are included or excluded in non-canonical ways, and it occurs in over 90% of human genes (146, 147). Aberrant splicing is well documented during infection and immune activation (148, 149), making AS a major source of proteome diversity with direct consequences for subcellular localization (Fig. 3B) (150).

Mitochondrial enoyl-CoA reductase (MECR) provides a striking example of how AS can repurpose a metabolic enzyme for antiviral defense. MECR is an essential component of the mitochondrial fatty acid synthesis pathway, generating acyl intermediates required for electron transport chain function, the TCA cycle, amino acid catabolism, and purine biosynthesis (151). Loss of MECR causes inborn metabolic errors in humans and embryonic lethality in mice (152). An AS product lacking the first exon and associated MTS produces an in-frame N-terminally truncated isoform that distributes throughout the cytoplasm and nucleus (153, 154). Remarkably, this relocalized isoform restricts influenza A virus by binding the viral polymerase and blocking ribonucleoprotein assembly and genome replication (154). By embedding an antiviral function within an energetically indispensable enzyme, the gene may be buffered against viral antagonism; disabling the antiviral isoform would simultaneously cripple mitochondrial metabolism.

Key apoptotic regulators also undergo AS that alters localization, affecting infection. BCL-2 family members produce AS isoforms that oppose the function of their canonical products (155–163). Although BCL-xL and MCL1 (introduced above) suppress apoptosis and BAX promotes it, the AS-derived isoforms BCL-xS and MCL1S are pro-apoptotic, and BAX-κ/ψ are anti-apoptotic. Infection promotes these AS events with BAX and BCL-X splicing dysregulated through modulation of the RNA-binding proteins RBM25 and SRSF6, respectively (164–166). IGRM and OPA1 are factors that balance mitochondrial fission and fusion, a function that is required for MAVS platforms. AS generates variants of all three proteins, IGRM, OPA1, and MAVS, with localization changes that dampen immune signaling (167–170). Finally, promyelocytic leukemia protein (PML), although predominantly nuclear, produces AS isoforms that relocalize proximal to mitochondria under stress conditions (171, 172).

#### Alternative translation initiation (ATrI)

Translation initiation positions a ribosome at the start codon of an mRNA. In canonical initiation, the small 40S ribosomal subunit assembles with eukaryotic initiation factor (eIF) proteins into a pre-initiation complex, binds the m7G 5′ cap via the eIF4F complex and the 3′ poly(A) tail via poly(A)-binding protein (PABP), and then scans the 5′ UTR for the AUG start codon (173). Context surrounding the AUG is critical: the Kozak sequence, comprising the six nucleotides upstream and one downstream of the AUG, strongly influences initiation efficiency (174). Non-canonical initiation modes become especially prominent during viral infection and cellular stress, including IRES-dependent initiation, *N*^6^-methyladenosine-dependent initiation, and eIF2-independent mechanisms (173). Because roughly 50% of mRNAs produce more than one protein through ATrI (175, 176), and N-terminal extensions or truncations directly determine mitochondrial targeting, ATrI represents a broad and underappreciated source of organellar proteome variation (Fig. 3C).

In a computational screening approach, Kazak et al. systematically identified non-mitochondrial proteins that could acquire mitochondrial localization through ATrI (177). The authors identified genes conserved between mouse and man that contained in-frame AUGs within the first 25% of the coding region and where there was no prior MTS annotated. This list was then filtered through three mitochondrial localization prediction tools to identify 126 putative mitochondrial-localizing products. More recently, Ly et al. evaluated the global impact of N-terminal aberrations directly on predicted protein localization (178). The authors used ribosome profiling data to identify initiation sites within the genes of HeLa cells and then used a localization prediction algorithm to determine whether an N-terminal extension or truncation would impact localization. The authors predicted that 125 genes encoded an N-terminal variant that would either gain or lose mitochondrial localization. It is worth noting that although both reports validated several of their predictions, showing localization changes due to ATrI, there are zero overlapping protein-coding genes across the two approaches.

ATrI also regulates mitochondrial targeting in the context of inflammation and viral infection. The gap junction protein connexin 43 (Cx43, encoded by GJA1) forms intercellular channels at the cell periphery and is linked to inflammatory disease through augmented release of pro-inflammatory molecules (179, 180). A mitochondrial pool of Cx43 participates in stress responses (181), and an ATrI isoform of GJA1, termed GJA1-20k, relocalizes to mitochondria under stress where it induces mitochondrial biogenesis and protects mice against ischemia-reperfusion injury (182, 183). Many viruses, including tumor-associated viruses such as human papillomavirus (HPV), target Cx43, although the connection between viral Cx43 modulation and the mitochondrial isoform remains unexplored (184). MAVS provides a second example of ATrI-mediated functional control, although localization remains mitochondrial. An N-terminally truncated isoform called miniMAVS retains mitochondrial anchoring through its conserved C-terminal transmembrane domain but lacks the caspase activation and recruitment domain (CARD), positioning it as a potential modulator of full-length MAVS signaling (185).

#### Translational recoding

In canonical translation elongation, the ribosome reads mRNA codons sequentially and terminates upon encountering one of three stop codons (UAA, UAG, or UGA). Recoding departs from this fidelity through three mechanisms: stop codon readthrough, which extends the C-terminus; frameshifting, in which the ribosome slips to an alternate ORF to produce a fusion protein; and bypassing, in which the ribosome skips internal sequence to produce an internally deleted product (186). General translation fidelity is high, with missense errors below 10⁻⁴ per codon, but readthrough and frameshifting reach 0.1–0.3 and 0.05–0.8 per codon, respectively, when favored by *cis*-acting mRNA structures and *trans*-acting factors (186). Recoding-mediated dual localization is conserved from fungi to animals, as shown for peroxisomal inorganic phosphatase (187). Likewise, machine learning applied to ribosome profiling data has recently identified sequence features that predict readthrough conservation from yeast to humans (188).

Most viruses use recoding to expand proteome diversity within compact genomes (189, 190). Viral infection globally reshapes the host translatome. During human cytomegalovirus (HCMV), vaccinia, human immunodeficiency virus (HIV), and influenza infection, the repertoire of translated mRNAs shifts substantially (48, 191–194). Japanese encephalitis virus (JEV) generates the protein NS1′ through ribosomal frameshifting of NS2A (195). NS1′ does not itself relocalize, but it limits mitochondrial immune signaling indirectly by inducing miR-22 expression, which suppresses MAVS (196).

Dual localization through translational readthrough is typified by two host proteins, MTCH2 and AGO1. MTCH2 is a mitochondrial insertase that facilitates the insertion of α-helical peptides into the OMM (197). *MTCH2* generates two additional proteins through stop codon readthrough, MTCH2x and MTCH2xx, whose expression is important to regulate MOMP (198). MTCH2x maintains mitochondrial localization, but MTCH2xx is distributed throughout the cytoplasm. The influenza A virus polymerase subunit PA interacts with MTCH2 and reduces viral polymerase activity (199). Given that PA shuttles between the nucleus and cytoplasm, MTCH2xx is the likely interacting partner. AGO1 is an Argonaute family protein involved in miRNA-mediated mRNA turnover (200). Its readthrough isoform, AGO1x, exerts dominant negative activity on the miRNA pathway (201). AGO1x relocalizes from the cytoplasm to the nucleus, but through an interaction with the mitochondrial enzyme polyribonucleotide nucleotidyltransferase 1 (PNPT1), it degrades dsRNA and attenuates interferon signaling and apoptosis (202, 203). Both MTCH2x/xx and AGO1x are broadly conserved, supporting the idea that encoding dual-localized immune regulators through readthrough confers a selective advantage (198, 203).

### Protein-based mechanisms of mitochondrial localization

Nearly all mitochondrial proteins are encoded by the nucleus, translated in the cytoplasm, and imported into the organelle. Under stress, retrograde signaling from mitochondria to the nucleus upregulates nuclear-encoded mitochondrial factors, including electron transport chain subunits (204–206). This bidirectional communication is being explored in the context of mitonuclear co-regulation to address how two genomes of distinct evolutionary origin and unequal copy number maintain stoichiometric balance across multi-protein complexes (207, 208). Viral-induced mitochondrial dysfunction may therefore paradoxically upregulate the very import machinery that licenses immune-active protein pools, creating a feedback loop with functional consequences for host defense. Regulation of mitochondrial import and export during infection can be driven by protease cleavage, PTM, or conformational changes. If a cellular protein reaches mitochondria through a defined pathway, viruses often exploit that same circuitry. Both host and viral proteins are often multifunctional, and protein-level regulation can generate distinct molecular pools with different fates. One form may traffic to mitochondria while an unmodified counterpart localizes to the nucleus or to viral replication factories (49, 209–211).

Viral mitochondrial targeting sequences closely mimic their host counterparts. They are typically around 20 amino acids long, positively charged, and predicted to form amphipathic α-helices (212–216). Most MTSs reside near the N-terminus, although internal MTSs have been identified. MTSs are loosely defined and degenerate enough to function across divergent species, as shown by studies of mammalian proteins expressed in yeast. Some viral proteins carry identifiable MTSs, such as hepatitis B virus Pol (217) and calicivirus NS3 (49), but many do not.

Mitochondria are sites of innate immune signaling platforms. Key to this signaling is MAVS, which requires mitochondrial trafficking prior to higher-order complex formation of the signalosome. MAVS is anchored to the mitochondrial OMM by a C-terminal transmembrane domain. Upon binding immunostimulatory RNAs, RLRs RIG-I and MDA-5 translocate to mitochondria to activate the adaptor protein MAVS. Following target RNA binding, RIG-I undergoes a structural rearrangement that releases its CARD domain, enabling RIG-I translocation to mitochondria and homotypic interaction with the CARD domain of MAVS (218). RLR activation and signaling result in TBK1- and IKK-mediated phosphorylation of MAVS at its pLxIS motif (219). pLxIS motifs are conserved motifs that are common in immune adaptor proteins, which are involved in the regulation of interferons and metabolism (220). Phosphorylation of MAVS facilitates interaction with the cytoplasmic transcription factor IRF3. Recruitment of IRF3 to mitochondria facilitates its interaction with TBK1 and subsequent IRF3 phosphorylation. Phosphorylated IRF3 translocates to the nucleus, where it activates its target genes, including type I interferons. MAVS recruits many additional proteins to mitochondria, in part, by leveraging specific host-encoded RNAs that contribute to the MAVS signalosome (221). Thus, the MAVS signalosome represents a relevant example to study the inducible recruitment, docking, and release of proteins and RNAs from a large complex attached to the outer mitochondrial membrane during infection (221).

#### Protease cleavage and mitochondrial localization

Cleavage of the N-terminal presequence is a defining step in the maturation of most resident mitochondrial proteins (222, 223). More broadly, protease cleavage is one of the most versatile regulatory mechanisms in cell biology: it can release an inhibitory domain, unmask a localization signal, target a protein for proteasomal degradation, or even activate a pathway through functional degradation (Fig. 3D), as seen for the NLRP1 inflammasome protein when engaged by pathogen-encoded antagonists (224, 225).

MAVS activity depends on its localization to the OMM, and viral or cellular proteases that cleave it from the membrane effectively disarm the pathway. The first such protease identified was NS3/4A from hepatitis C virus (HCV), whose amphipathic helix is required for mitochondrial targeting and MAVS cleavage (226, 227). Coxsackievirus B 3C protease and Enterovirus 71 2Apro subsequently joined this list (228, 229). Host caspases also cleave MAVS during apoptosis, underscoring that cellular and viral strategies for disabling the adaptor overlap considerably (230–232).

Host protease activity also drives mitochondrial localization changes. Apoptosis-inducing factor (AIF) normally resides in the inner mitochondrial membrane space, where it functions as an NADH oxidase (233). It is synthesized as a 67 kDa precursor with an N-terminal MTS; import and processing yield a 62 kDa mature form. During stress or infection by HSV-1 and HIV-1, the host protease calpain I cleaves AIF to 57 kDa, and the truncated protein is released through MOMP pores formed by pro-apoptotic BCL-2 proteins (234–238). AIF then traffics to the nucleus via two nuclear localization sequences (NLS), where it drives chromosome condensation, large-scale DNA fragmentation, and release of proapoptotic stimuli including cytochrome c (239, 240). AIF demonstrates the general principle that protease cleavage of a host factor can remove an MTS to re-license a protein for a different subcellular localization and function during stress. In inflammasome-activated cells, caspase-1 cleaves gasdermin D (GSDMD), relocalizing N-GSDMD to both the plasma membrane and the OMM to execute pyroptosis and necroptosis, respectively (241, 242). DNA topoisomerase 3-α (TOP3A) carries both an N-terminal MTS and a C-terminal nuclear localization sequence (NLS). In the nucleus, TOP3A resolves Holliday junctions and regulates DNA topology as part of a larger protein complex, while the mitochondrial isoform deconcatenates replication intermediates during mtDNA replication (243, 244). Mitochondrial processing peptidase (MPP) removes the TOP3A C-terminus upon import, an event that adapts its biochemical activity for the mitochondrial substrate. The result is two functionally distinct pools of the same protein, shaped not just by localization but by the proteolytic processing that occurs at the destination.

A spatial division of labor for multifunctional viral proteins can be regulated by proteolysis. Hepatitis A virus (HAV) exhibits this through the 3ABC precursor (211). Many viruses express polyproteins that are processed into functional subunits by serial cleavage; in HAV, 3A encodes a transmembrane domain, and 3C encodes a protease. The intact 3ABC precursor localizes to mitochondria via the transmembrane domain in 3A, where 3C-mediated cleavage of MAVS suppresses innate immune signaling. Subsequent self-cleavage by 3C then releases 3ABC from the membrane, freeing the protease domain for other functions. The domain arrangement of an MTS fused to a protease domain implies tight temporal regulation, since suppression of cell-intrinsic defenses must precede productive viral replication. A phylogenetically distant example comes from the plant virus Grapevine leafroll-associated virus 3 (GLRaV3), which exploits the OMM to form viral replication compartments (245). This virus appears to exploit the OMM to form virus replication compartments. Its ORF1a polyprotein, 2,237 amino acids in length, encodes a papain-like protease, methyltransferase, AlkB domain, and helicase (246). ORF1a localizes to mitochondria via an internal MTS near residue 829, and this MTS comprises an amphipathic helix followed by a downstream transmembrane segment beginning around residue 1069. Cleavage within this region appears to release portions of ORF1a from the membrane, suggesting that protease-mediated mitochondrial liberation is not restricted to animal viruses but operates across biological kingdoms.

Proteolytic processing of viral polyproteins can itself generate the mature proteins that traffic to mitochondria. Following signal peptidase cleavage of the HCV polyprotein at the ER, the core protein is directed to the OMM by a 10 amino acid C-terminal motif (247). Core encapsidates the viral RNA genome and also perturbs mitochondrial function, releasing reactive oxygen species from the electron transport chain and promoting cytochrome c release through interaction with MCL1 via a BH3 domain (248, 249). Processing of the Dengue virus (DENV) polyprotein similarly produces NS3pro (180 amino acids) and NS3pro-helicase (464 amino acids), both of which localize to the mitochondrial matrix via N-terminal MTSs, where DENV NS3 cleaves the mtHSP70 co-chaperone GrpEL1 (250). These examples show that polyprotein processing routes viral proteins to mitochondria to unlock immune-modulatory function.

#### Post-translational modifications and mitochondrial trafficking

Post-translational modifications regulate protein localization by reversibly altering protein charge, conformation, and interaction surfaces (Fig. 3E). Their roles span signal transduction, development, gene expression (235), and viral infection (82, 251–255), development, gene expression, and viral infection. A quintessential example is JAK/STAT signaling. JAK/STAT proteins are core facets of many immune signaling pathways, and their activation involves a series of PTMs, mainly phosphorylation, which induce translocation of the STAT transcription factors to the nucleus to upregulate expression of target genes (254, 256–258).

Phosphorylation within an MTS can directly block mitochondrial import, with consequences for both immune signaling and viral infection. TFAM, described above, is associated with mtDNA depletion from mitochondria when its MTS is phosphorylated, preventing its mitochondrial import (135). The ISG *CNP* (2′,3′-cyclic nucleotide-3′-phosphodiesterase), specifically its isoform CNP2, demonstrates the same principle with an antiviral protein. CNP2 mitochondrial import is blocked by protein kinase C (PKC)-mediated phosphorylation of its MTS (259). CNP is implicated in the inhibition of HIV-1 (260, 261) and HBV (262). In both cases, phosphorylation functions as a reversible switch that controls access to the organelle rather than eliminating the protein altogether.

Phosphorylation of an MTS also governs the localization of viral proteins. pUL37x1, the HCMV-encoded viral mitochondria-localized inhibitor of apoptosis (vMIA), localizes to mitochondria via its own MTS to counteract BAX-mediated apoptosis (263–266). It also localizes to the ER, where it antagonizes calcium storage and PKR-like ER kinase (PERK) activity (75, 267). The balance of pUL37x1 between these compartments is controlled by a conserved PKC phosphorylation site; phosphomimetic mutations shift it to the ER, while alanine substitutions lead to mitochondrial enrichment (268). A single reversible phosphorylation event thus determines which compartment this multifunctional effector occupies. Vaccinia virus F17 provides a second viral example of phosphorylation-dependent mitochondrial targeting. F17 is a morphogenesis protein that localizes to mitochondria when phosphorylated and to viral factories when unphosphorylated (210, 269). Phosphorylated F17 specifically associates with mitochondria that are actively leaking mtDNA, where it antagonizes the metabolic regulator mTOR to suppress cGAS-driven ISG responses (270). The opposing localizations of the two F17 forms suggest tight coupling between phosphorylation state and the specific mitochondrial context that F17 evolved to counteract. Across these examples, dynamic populations of viral proteins move to mitochondria via PTMs at functionally precise moments.

Beyond phosphorylation, acetylation also promotes mitochondrial import of immune factors like STAT3. The transcription factor STAT3 is activated by key responses, such as IL-6 and growth factor signaling, and is imported into mitochondria (271). STAT3 mitochondrial import was discovered for its role in promoting Ras-mediated transformation. Interestingly, cellular Ras was initially discovered due to viral homologs encoded by oncogenic viruses (272). Following their phosphorylation, STAT3 and STAT proteins translocate to the nucleus, where they activate genes controlling immunity and infection outcomes. Both the STAT3 N-terminus and the linker region are important for mitochondrial import (273). Experimental evidence shows insulin signaling promotes STAT3 N-terminal acetylation that drives mitochondrial import (274). Import seems to be also mediated, in part, by chaperone activity of the Complex I ETC subunit GRIM-19/NDUFA13 (275). In mitochondria, STAT3 promotes OXPHOS (271, 276).

Looking ahead, phosphorylation of mitochondrial proteins represents fertile but largely unexplored territory in the context of infection. Most proteins in MitoCarta carry evidence of phosphorylation; however, the functional relevance of the vast majority of these events is unknown (277). Notably, many phosphorylation sites cluster in or near MTSs, raising the possibility that regulated import is far more widespread than currently appreciated. The broader PTM landscape during infection is also increasingly rich, and systematic mapping of how modifications alter protein localization in infected cells is likely to unearth mechanistic surprises (79, 278).

#### Mitochondrial localization and structural changes

The intra- and extracellular microenvironment is extensively remodeled during infection, but the consequences for protein localization remain incompletely defined (81, 279). Conformational changes driven by protein-protein interactions or stress cues represent an underappreciated mechanism by which cytosolic proteins acquire mitochondrial targeting (Fig. 3F).

BAX illustrates the principle of structural control of mitochondrial localization. Normally cytosolic and pro-apoptotic, BAX is activated by interactions with BH3-only proteins, triggering oligomerization and translocation to mitochondria (280). In response to stress cues, the cytosolic pro-apoptotic BAX protein interacts with BCL-2 homology 3 (BH3)-only proteins, which results in BAX oligomerization and targeting to mitochondria. BAX is activated by a BH3 domain in IRF3, which causes both to co-translocate to the OMM (281). There, structural rearrangement drives BAX insertion and complex formation, producing pores that cause MOMP and release of cytochrome c and other mitochondrial DAMPs (282, 283). BAX activity shapes the outcome of influenza A and Sindbis virus infections and is antagonized by vMIA/pUL37x1 (264, 284, 285).

Calicivirus NS3 undergoes a structural switch to toggle between housekeeping and lethal functions. NS3 is organized with an N-terminal MTS, followed by a 4-helical bundle (4HB) derived from the necroptotic effector MLKL and then an NTPase domain (49). In its housekeeping role, NS3 supports viral replication through NTPase, helicase, and RNA chaperoning activities (286, 287). During egress, however, NS3 drives mitochondrial permeabilization via its 4HB domain, which is highly toxic, suggesting strict temporal regulation. The nature of the conformational trigger that shifts NS3 from replication factories to mitochondria is not yet fully understood. This highlights how structural dynamics enable single proteins to occupy mitochondria at one stage of infection while supporting viral replication at another.

### Evolutionary mechanisms regulating mitochondrial localization

Mitochondria are evolutionary products of endosymbiosis, and the organelles remain physically and genetically dynamic (288). Over evolutionary time, nearly all genes originally encoded in the mitochondrial ancestor have transferred to the nuclear genome; however, their protein products continue to function in mitochondria while often acquiring extra-mitochondrial roles (34, 178, 289). Given their fundamental roles in homeostasis, stress adaptation, and host defense, mitochondria must therefore accommodate ongoing evolutionary pressures from viruses (9, 73, 205).

Gain of an MTS or loss of its function is among the most consequential mutations possible within a single step. The underlying mechanisms include coding sequence gains or losses affecting MTS-encoding exons, changes in splicing that alter MTS exon usage, and mutations that modify the start codon (290) or a PTM site governing import (Fig. 3G). Gene duplication further expands the space of possible outcomes, allowing the parental and daughter copies to diverge independently toward mitochondrial and non-mitochondrial fates (291). These innovations are not necessarily stable: MTS gains and losses can cycle repeatedly over evolutionary time.

Species-specific differences in mitochondrial targeting have direct consequences for immune signaling. The DNA sensor cGAS localizes to mitochondria in a species-specific manner, and this variation correlates with differences in self-DNA reactivity (292). cGAS senses cytosolic mtDNA as well as viral DNA, and sensing of infection-mediated released mtDNA contributes to interferon production, particularly during RNA virus infection (54, 55). Differences in cGAS-mitochondria association between humans and mice have practical implications for interpreting experimental results across model systems.

Species-specific differences also impact RLR/MAVS signaling outcomes. For example, rapid evolution of primate MAVS at the HCV NS3 protease cleavage site tips MAVS activation and consequently HCV replication due to differential cleavage susceptibility (293). Relatedly, both RIG-I and MDA-5 display signatures of adaptive evolution in mammals, which seem ongoing for RLRs in human populations (294, 295). Extreme evolutionary innovations for factors recruited to mitochondria during infection are highlighted by the loss of RIG-I in tree shrew, which may be compensated by adaptations in MDA-5 (296). Recurrent loss of RIG-I seems to be unexpectedly common, given its vital role in host defense. Specifically, RIG-I has been lost in bird genomes repeatedly, including chickens. These cases are particularly unexpected, given established roles for RIG-I in sensing influenza A virus (297, 298). Historically, genetic loss is viewed as a hallmark of dispensable functions. However, in the context of host-virus interactions, these turnover events may actually represent a signature of vital host determinants, pivotal host-virus interfaces, and even essential biological and species-specific innovations (299–306).

Viruses have also exploited horizontal gene transfer to acquire genes encoding mitochondrial activities. Several large DNA viruses carry host-derived electron transport chain factors and TCA cycle enzymes (43, 307). Norovirus NS3 is a particularly striking case. As described above, NS3 acquired an N-terminal MTS immediately upstream of an MLKL-derived pore-forming domain, retargeting a necroptotic cell death effector to drive mitochondrial permeabilization and viral egress (49, 308, 309). The necessity of the NS3 MTS for this activity demonstrates how a single targeting sequence acquisition can redirect an existing biochemical function toward a completely new subcellular outcome. The NTPase domain of NS3 shares homology with NTPases encoded by numerous other viruses, raising the possibility that uncharacterized N- or C-terminal extensions on other viral NTPases represent unrecognized targeting sequences. This evolutionary process appears ongoing in nuclear genomes as well, where alternative start codon usage drives proteome diversification and generates multiple protein pools from a single locus (178).

## CONCLUSIONS

The studies reviewed here collectively show that mitochondrial protein trafficking is not a static feature of cell biology but a dynamic, infection-sensitive process with direct consequences for host defense. Cells and viruses repurpose canonical mitochondrial factors through alternative transcription, splicing, translation, proteolysis, and post-translational modification to execute immune functions that are spatially and temporally distinct from their housekeeping roles.

Several questions at the frontier of this field concern the mitochondrial proteome itself and how it fluctuates during infection. The co-evolutionary history of mitochondrial (α-proteobacterial), host, and viral genomes together with recurrent gene flow between them is a lens through which to view proteins with dual-localization functions (24). Mitochondrial membrane potential is essential for the import of many proteins, and perturbation of the electron transport chain, a hallmark of virally infected cells, impairs protein import (310). Which proteins fail to reach mitochondria during infection, and what the downstream consequences of their cytosolic accumulation are, represent largely uncharted territory. Defective mitochondrial import activates the mitoCPR (mitochondrial compromised protein import response), a transcriptional program that upregulates import machinery and mitochondrial quality control factors (311). Whether this mitoCPR is engaged during viral infection and whether it reshapes immune-active protein pools are open questions. Compositional heterogeneity adds another layer of complexity: even within a single cell, individual mitochondria can differ substantially in their functional state due to stress (312). A comprehensive accounting of which proteins traffic to which mitochondrial subpopulations and how infection shifts this distribution will be necessary to fully understand how the organelle integrates metabolic and immune demands.

Investigating these questions requires methodological advances as much as conceptual ones. Live-cell imaging platforms capable of tracking protein localization dynamics are critical. To better understand mitochondrial composition, it will be important to pair mitochondrial flow cytometry with imaging, proteomics, and deep learning (242, 310, 313). Complementing these approaches, computational tools for predicting mitochondrial targeting sequences and transmembrane domains, such as DeepLoc2, TMHMM, and MitoFates, among others, provide tractable means of generating testable hypotheses from proteome-wide data sets (314–316). Applying these tools to ribosome profiling, proteomics, and alternative isoform catalogs from infected cells has the potential to identify a new wave of moonlighting factors not yet connected to mitochondrial biology.

The field is also poised to benefit from systematic analyses of epigenetic and epitranscriptomic regulation, together with metabolic context. Infection-induced changes in RNA methylation, histone modification, and chromatin accessibility can alter which isoforms are expressed, in what abundance, and to where they localize (317, 318). Additionally, standard high-glucose culture conditions modulate mitochondrial membrane potential in ways that likely do not reflect virally infected cells *in vivo*, where mitochondrial dysfunction is routine (73). Altered metabolic states increasingly appear to prime or dampen immune responses (66), and it is plausible that failed or reduced mitochondrial import under such conditions contributes to phenotypic observations. Careful attention to metabolic context both in experimental design and in the interpretation of published data will be important for reconciling conflicting localization reports in the literature.

Finally, the principles described here carry translational implications. The specificity of MTSs, and the diverse mechanisms by which they can be gained, lost, or conditionally activated, present opportunities for the rational design of antivirals that disrupt critical viral-mitochondrial interactions or block the redistribution of host factors exploited by pathogens. Similarly, MTS-based delivery strategies could be leveraged to direct therapeutic payloads to mitochondria in infected cells with spatial precision (319). As the list of proteins with condition-dependent mitochondrial localization continues to grow, so too does the toolkit for intervening in the processes they control.

## SUPPLEMENTAL MATERIAL

Table S1 jvi.01746-25-s0001.xlsx Full gene lists to identify mitochondrion-localized proteins with putative immune function.

ASM does not own the copyrights to Supplemental Material that may be linked to, or accessed through, an article. The authors have granted ASM a non-exclusive, world-wide license to publish the Supplemental Material files. Please contact the corresponding author directly for reuse.
